# S100A9 as a shared biomarker and mediator of metabolic dysfunction in peripheral artery disease and sarcopenia

**DOI:** 10.3389/fgene.2026.1874960

**Published:** 2026-07-02

**Authors:** Yaming Guo, Wenxin Zhao, Hai Feng, Yongjun Li

**Affiliations:** 1 The Key Laboratory of Geriatrics of NHC, Institute of Geriatric Medicine, Beijing Hospital, National Center for Gerontology, National Clinical Research Center for Gerontology, Chinese Academy of Medical Sciences & Peking Union Medical College, Beijing, China; 2 Department of Vascular Surgery, Beijing Friendship Hospital, Capital Medical University, Beijing, China; 3 Medical School, University of Chinese Academy of Sciences, Beijing, China

**Keywords:** biomarker, inflammation, PAD, S100A9, sarcopenia

## Abstract

**Backgrounds:**

Peripheral artery disease (PAD) frequently causes to persistent functional impairment in skeletal muscle even after successful revascularization, implicating non-ischemic pathological mechanisms. Sarcopenia, a myopathy characterized by progressive loss of muscle mass, strength, and function—shares these non-ischemic features and affects approximately one-third of PAD patients, yet the molecular basis of their comorbidity remains poorly defined.

**Methods:**

Three transcriptome datasets (GSE120642, GSE181930, and GSE226151) were included in the analysis, covering skeletal muscle samples from peripheral artery disease (PAD) and sarcopenia. Weighted gene co-expression network analysis (WGCNA) was performed independently for each disease cohort, followed by parallel feature selection using three machine learning algorithms (LASSO, Random Forest, and Boruta) to identify shared diagnostic biomarkers. Immune cell infiltration was deconvoluted using CIBERSORT. Drug-gene interaction analysis was conducted via DGIdb. The functional role of the lead candidate S100A9 was validated by untargeted metabolomic profiling of C2C12 myoblasts treated with recombinant S100A9.

**Results:**

Seventy-six overlapping disease-associated genes were identified from WGCNA, and five core diagnostic biomarkers—BCKDHB, PIM1, JAML, NFE2, and S100A9 — were selected through three-way machine learning consensus. Enrichment analyses revealed shared involvement of innate immune activation, granulocyte infiltration, and branched-chain amino acid (BCAA) catabolism. CIBERSORT deconvolution confirmed elevated neutrophil abundance as a convergent immune feature of both diseases. Metabolomic profiling demonstrated that recombinant S100A9 disrupted nucleotide and energy homeostasis, induced mitophagy dysregulation, and promoted oxidative stress in C2C12 myoblasts. DGIdb screening identified Paquinimod, a selective S100A9 inhibitor with Phase II clinical safety data, as a candidate for therapeutic repositioning.

**Conclusion:**

This study reveals that upregulation of skeletal muscle inflammation and abnormal branched-chain amino acid metabolism may be common features of PAD and sarcopenia. BCKDHB, PIM1, JAML, NFE2, and S100A9 were identified as common diagnostic biomarkers, and metabolomics further confirmed that S100A9 may be a potential intervention target.

## 1 Introduction

Peripheral artery disease (PAD) is a disabling vascular disease whose global burden is significantly increasing with population aging. It is estimated that over 230 million people worldwide suffer from this disease (Conte et al., 2019). Current treatment strategies center on surgical intervention or endovascular revascularization to restore macroscopic blood flow. However, even after technically successful revascularization, patients show marked heterogeneity in wound healing and function recovery of skeletal muscle (Park et al., 2022), revealing a persistent discordance between restored blood flow and clinical outcomes.

Skeletal muscle is the primary tissue injured in PAD, and its tissue immune microenvironment is a critical determinant of regenerative capacity (Yuan and Larsson, 2023). Although ischemia initiates tissue damage, structural abnormalities in ischemic skeletal muscle often resist complete reverse after revascularization (Wilburn et al., 2024). Moreover, substantial evidence from non-ischemic diseases demonstrates that skeletal muscle remodeling is governed by intrinsic, non-hemodynamic processes, including microcirculatory dysfunction, impaired mitochondrial metabolism, and defective regeneration (Chen et al., 2023). For instance, muscle degeneration in sepsis, cancer, and other systemic conditions is driven by chronic inflammation and metabolic disorders rather than hypoperfusion (Argilés et al., 2014; Hyatt and Powers, 2021; Wang et al., 2025). These factors may similarly contribute to skeletal muscle injury in PAD (Hyatt and Powers, 2021; Koike et al., 2022).

Sarcopenia, a myopathy characterized by progressive loss of skeletal muscle mass, strength, and function, provides an important conceptual framework for understanding the non-ischemic contributions (Guo et al., 2024; Cruz-Jentoft et al., 2019). Epidemiological studies indicate that sarcopenia affects approximately one-third of PAD patients, a prevalence substantially higher than in age-matched controls, and is independently associated with worse limb outcomes and increased mortality (Pizzimenti et al., 2020; Addison et al., 2018). Critically, sarcopenia-associated muscle degeneration is not simply a consequence of reduced perfusion. Even without overt ischemia, age-related muscle loss is accompanied by microvascular rarefaction, impaired angiogenic signaling and diminished regenerative potential (Duscha et al., 2020; Prior et al., 2016; Ma et al., 2025). Accordingly, sarcopenia is not merely a downstream sequela of hypoperfusion in PAD, but an autonomous and interactive pathological determinant that shapes heterogeneity in skeletal muscle remodeling and functional recovery. Despite this conceptual advance, the mechanisms governing the comorbidity between PAD and sarcopenia remain largely undefined.

Here, we systematically characterize the immune landscape of PAD–sarcopenia comorbidity through integrative transcriptomic analysis, identify key regulatory molecules, and provide a molecular rationale for combined vascular–muscle therapeutic strategies.

## 2 Methods

### 2.1 Data preprocessing and differential gene identification

**FIGURE 1:**
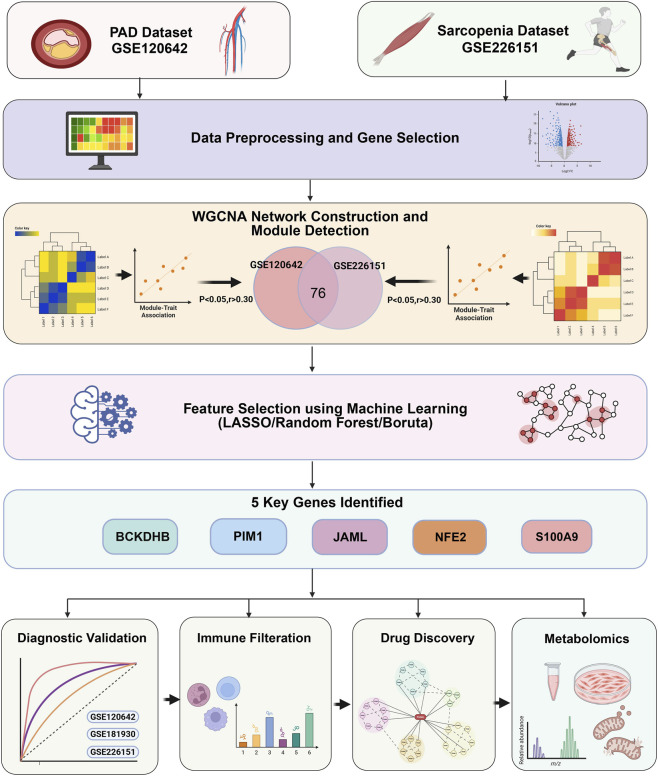
Schematic overview of the cross-disease analysis and validation framework (Created with BioRender.com).

This study analyzed transcriptome datasets GSE120642, GSE181930, and GSE226151 (https://www.ncbi.nlm.nih.gov/geo/). The comprehensive experimental design, sequential bioinformatic pipeline, and downstream functional validation workflow of this study are schematically illustrated in Figure 1. To maintain statistical rigor and avoid artificial technical bias, these datasets were processed individually and treated as separate cohorts with distinct functional roles in our workflow rather than being pooled or merged upstream. Specifically, for the PAD arm, GSE120642 (comprising 36 PAD cases and 15 controls) was designated as the discovery cohort for the initial differential expression identification and WGCNA network construction. Conversely, GSE181930 (comprising 13 PAD patients and 7 controls) was strictly reserved as a completely blind, independent external validation cohort to evaluate the diagnostic generalizability of the final biomarker signature. For the sarcopenia arm, GSE226151 (including 20 sarcopenia patients and 40 controls) was analyzed independently as the target myopathy cohort. All samples across these three cohorts were derived from human skeletal muscle tissue. For each individual dataset, background correction, normalization, and expression calculation were conducted autonomously within their respective platform architectures. Principal Component Analysis (PCA) was performed independently for each cohort to evaluate sample clustering and detect potential intra-dataset outliers.

Expression matrices for each dataset underwent standardized preprocessing. First, transcripts were mapped to gene symbols according to the platform annotation files. For genes with multiple probes, the median expression value was taken as the representative expression level. For RNA-seq data, the raw count matrix was converted to counts per million (CPM) and then log2-transformed using log2(CPM+1) to improve data distribution and meet the assumptions of subsequent linear model analyses. To ensure cross-cohort comparability, only the intersection of common gene symbols across all platforms was identified.

Differential expression analysis was performed independently for each cohort using the DEseq2 package. Genes with a significance threshold of p value < 0.05 and LogFC> 0.5were considered significantly differentially expressed. The Benjamini–Hochberg method was applied to control the false discovery rate for multiple comparisons.

### 2.2 Weighted gene co-expression network analysis

To identify functional co-expression modules associated with disease pathologies, weighted gene co-expression network analysis (WGCNA) was performed independently for the PAD discovery cohort (GSE120642) and the sarcopenia cohort (GSE226151) using the WGCNA R package (Langfelder and Horvath, 2008). For each preprocessed expression matrix, the top 30% of genes with the highest variance were selected for network construction. A soft-thresholding power (β), satisfying the approximate scale-free topology criterion (R2>0.85), was individually determined from a range of 1–20, resulting in an optimal β = 9 for the PAD dataset and β = 7 for the sarcopenia dataset. Subsequently, the respective correlation matrices were transformed into adjacency matrices and then into Topological Overlap Matrices (TOM). Average-linkage hierarchical clustering and dynamic tree cutting were performed to identify co-expression modules, with a merge cut height of 0.3 and a minimum module size of 50. Module eigengenes (MEs) were then calculated for each module. To identify clinical significance, Pearson correlation analysis was conducted between MEs and the respective disease status within each dataset. Modules with the highest correlation coefficient (r ≥ 0.3) and statistical significance (P < 0.05) were defined as the key disease-related modules. Within these key modules, gene significance (GS) and module membership (MM) were computed. Finally, instead of conducting a consensus WGCNA, a downstream intersection strategy was applied to identify shared drivers; the overlapping gene list was obtained by intersecting the genes from the significant positively correlated modules of both independent networks for subsequent machine learning feature selection.

### 2.3 Functional enrichment PPI network analysis

Functional enrichment analysis was performed using the ClusterProfiler R package. Gene Ontology (GO) terms (Biological Process, Cellular Component, Molecular Function) and KEGG pathways were evaluated. The Benjamini–Hochberg method was applied, with a false discovery rate (FDR) < 0.05 considered significant. Results were visualized using bubble or bar plots. A protein-protein interaction network was constructed via GeneMANIA (https://genemania.org/) to identify potential core genes (Warde-Farley et al., 2010).

### 2.4 Machine learning-based diagnostic biomarker selection

To identify key feature genes with diagnostic potential from comorbidity-related candidate genes, this study constructed an integrated machine learning feature selection process based on the intersection genes of key modules identified by WGCNA. Three algorithms—LASSO-logistic regression, Random Forest, and Boruta—were used in parallel to screen candidate genes (Kursa and Rudnicki, 2010; Tibshirani, 1996; Breiman, 2001). Specifically, the LASSO-logistic regression model determined the optimal penalty parameter λ through 10-fold cross-validation and retained genes with non-zero regression coefficients at λ; the Random Forest algorithm assessed gene importance based on Mean Decrease Gini, selecting top-ranking genes; and the Boruta algorithm retained genes deemed confirmed by comparing the true variables with shadow features generated by random permutations. Finally, the screening results of the three algorithms were integrated, and the intersection genes were selected as core candidate biomarkers for subsequent model construction and performance evaluation.

### 2.5 CIBERSORT immune cell infiltration analysis

To assess the immune microenvironment of PAD and sarcopenia in skeletal muscle tissue, this study employed the CIBERSORT algorithm to infer immune cell infiltration profiles from the transcriptome expression matrix (Newman et al., 2015). The standardized gene expression matrix was input into CIBERSORT with the LM22 immune cell signature matrix, and 1,000 permutations were performed to obtain robust statistical estimates (Conning-Rowland et al., 2025). To ensure reliability, only samples with a CIBERSORT output P < 0.05 were retained for subsequent analyses. The resulting relative abundance matrix of immune cells was used for between-group comparisons, with multiple testing correction performed using the Benjamini–Hochberg method. Furthermore, to investigate the potential regulatory relationship between core genes and the local immune status, Spearman correlation analysis was conducted based on the expression levels of core genes and the relative abundance of each immune cell type.

### 2.6 Drug candidate screening

To screen potential drug candidates targeting core pivot genes, this study submitted the identified core genes to the Drug-Gene Interaction Database (https://www.dgidb.org) for analysis. Using the built-in interaction annotation function, information on small molecule compounds that can target these core genes was extracted. Through intersection analysis of candidate drugs corresponding to different genes, common drug candidates that can act simultaneously on all core pivot genes were finally screened.

### 2.7 Metabolomics analysis

C2C12 mouse skeletal muscle myoblasts were cultured in high-glucose DMEM (4.5 g/L) with 10% fetal bovine serum and 1% penicillin-streptomycin at 37 °C in 5% CO_2_. At 70%–80% confluence, cells were treated with either PBS (control) or recombinant mouse S100A9 (1 μg/mL) for 48 h. After washing with ice-cold PBS, cells were lysed in 80% methanol/water (v/v) at −20 °C for 30 min. The lysate was centrifuged at 12,000 × g for 10 min at 4 °C, and the supernatant was lyophilized. Dried samples were reconstituted in water/methanol (50:50, v/v) and analyzed by LC-MS/MS using a C18 column with a gradient mobile phase of 0.1% formic acid in water and acetonitrile. Mass spectrometry was performed in positive and negative ion modes with data-dependent acquisition (DDA). Raw data were processed using Compound Discoverer for peak detection, alignment, and normalization. Principal component analysis (PCA) and partial least squares discriminant analysis (PLS-DA) were used to assess metabolic differences. Differentially expressed metabolites were identified using criteria of |Fold Change|≥ 2, VIP > 1, and P < 0.05, and then mapped to KEGG pathways and enriched via MetaboAnalyst.

### 2.8 Statistical analysis

All statistical analyses were performed in R and GraphPad Prism. Continuous variables were first subjected to the Shapiro–Wilk normality test; those conforming to a normal distribution were expressed as mean ± standard deviation, and comparisons between groups were performed using the independent samples t-test; Spearman correlation was used for correlation analysis. Multiple test correction for differential expression analysis was performed using the Benjamini–Hochberg method to control for false discovery. The performance of the machine learning model was evaluated using the area under the ROC curve. Unless otherwise specified, all tests were two-tailed, and p < 0.05 was considered statistically significant.

## 3 Results

### 3.1 Identification of DEGs in PAD and sarcopenia

**FIGURE 2:**
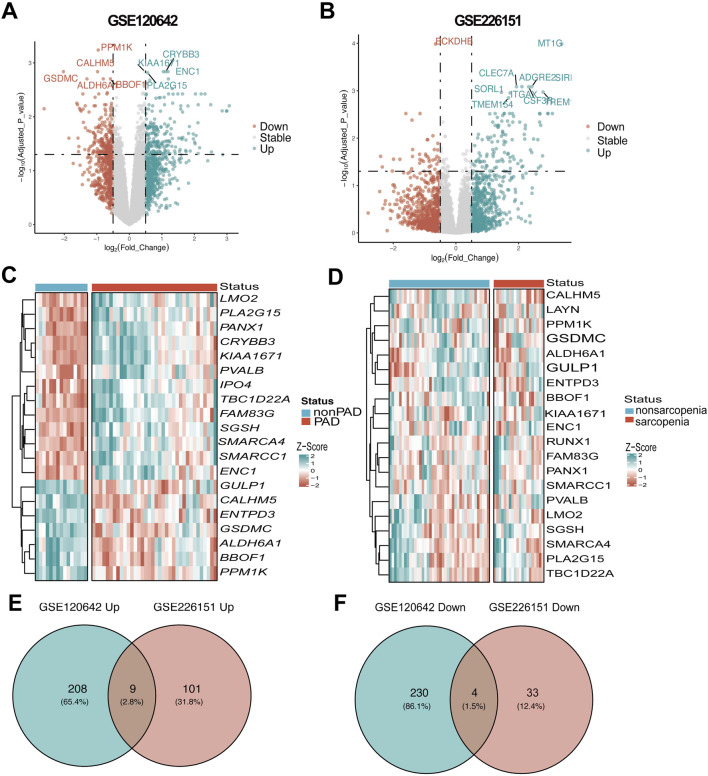
Identification of differentially expressed genes (DEGs). **(A)** Volcano plot showing the distribution of DEGs in the PAD cohort GSE120642. **(B)** Volcano plot showing the distribution of DEGs in the sarcopenia cohort GSE226151. **(C)** Heat map of the top 20 most significantly expressed DEGs in the PAD cohort GSE120642. **(D)** Heat map of the top 20 most significantly expressed DEGs in the sarcopenia cohort GSE226151. **(E,F)** Venn diagrams of upregulated and downregulated genes in PAD and sarcopenia.

In GSE120642 we initially identified 451 differentially expressed genes (DEGs), including 217 upregulated genes and 234 downregulated genes. Volcano plots were used to visualize the overall distribution of these DEGs, and heatmaps were further used to present these significantly upregulated and downregulated genes (Figures 2A,C). Among them, CRYBB3, KIAA1671, ENC1, and PLA2G15 showed significant upregulation in the PAD samples, while PPM1K, GSDMC, and ALDH6A1 showed significant downregulation. In the sarcopenia-related dataset GSE226151, a total of 147 DEGs were identified, including 110 upregulated genes and 37 downregulated genes. Volcano plots showed that multiple immune and inflammation-related genes were significantly upregulated in sarcopenia samples, with MT1G being one of the most significantly upregulated genes (Figures 2B,D). Cross-analysis of DEGs in PAD and sarcopenia datasets revealed 13 common differentially expressed genes between the two diseases. Among them, four genes were consistently downregulated, and nine genes were consistently upregulated (Figures 2E,F).

### 3.2 WGCNA analysis of PAD and sarcopenia

**FIGURE 3:**
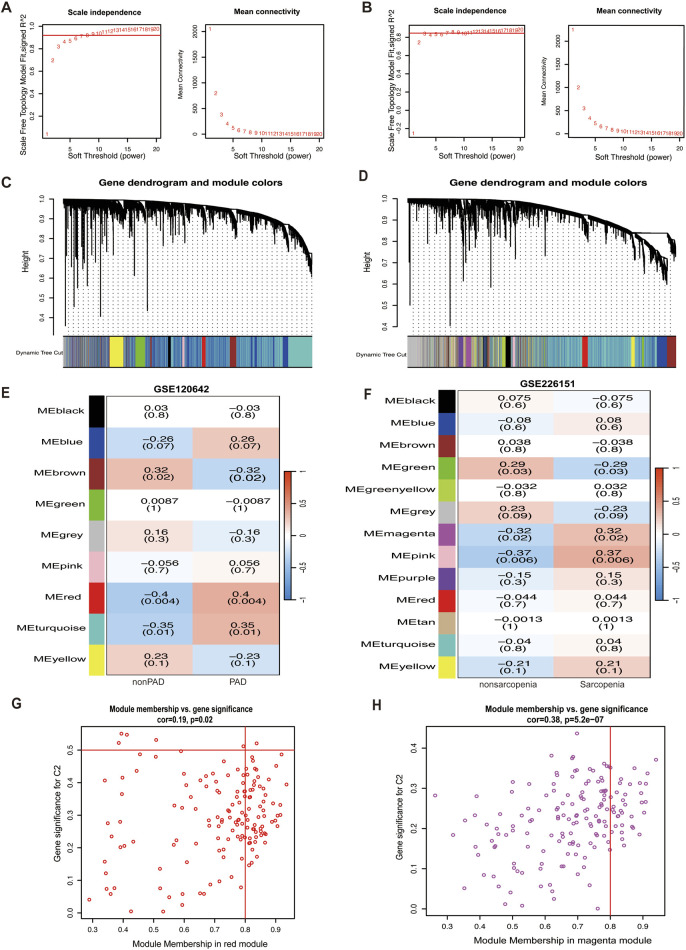
Weighted gene co-expression network analysis (WGCNA) of PAD and sarcopenia. **(A,B)** Mean connectivity of soft thresholding powers in the respective cohorts. **(C,D)** Cluster dendrograms of genes. **(E,F)** Heat maps of correlations between module eigengenes and clinical traits in PAD and sarcopenia. **(G,H)** Associations between significant modules and key genes in PAD and sarcopenia.

To investigate the disease-specific co-expression networks, WGCNA was performed independently on the expression matrices of the PAD discovery cohort (GSE120642) and the sarcopenia cohort (GSE226151). Under the approximate scale-free topology criterion, the optimal soft-thresholding powers (β) for the PAD and sarcopenia datasets were determined to be 9 and 7, respectively (Figures 3A,B). Module clustering via dynamic tree cutting identified 9 distinct co-expression modules in the PAD network and 13 modules in the sarcopenia network (Figures 3C,D).

To evaluate the clinical relevance of these networks, module–trait relationship analysis was conducted separately against the respective disease status of each independent cohort. This analysis revealed that the pink and magenta modules exhibited the most significant positive correlation with sarcopenia status (r = 0.37 and r = 0.32, respectively). Meanwhile, the red module showed the strongest positive correlation (r = 0.40) with PAD status (Figures 3E,F). Notably, within these key modules, a robust association was observed between gene significance (GS) and module membership (MM), yielding a correlation coefficient of 0.38 for the sarcopenia key modules and 0.19 for the PAD key module (Figures 3G,H), verifying that the hub genes within these modules are highly representative of their respective disease traits.

### 3.3 Enrichment analysis of common driver genes in PAD and sarcopenia

**FIGURE 4:**
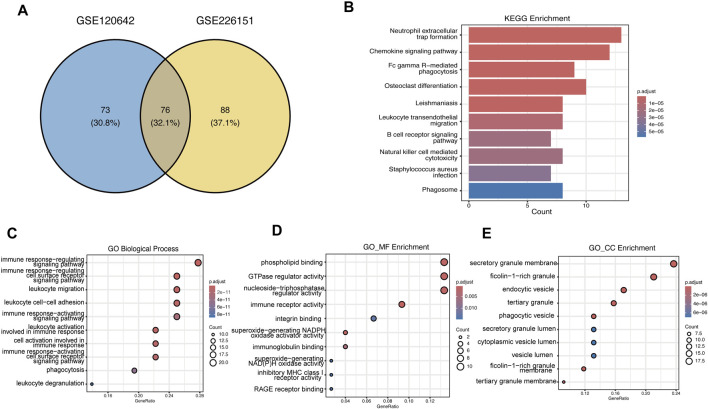
Enrichment analysis of co-expressed genes. **(A)** Overlapping genes between WGCNA modules from GSE120642 (PAD) and GSE226151 (sarcopenia). **(B)** KEGG enrichment analysis results. **(C–E)** GO enrichment analysis results (BP, CC, MF).

Next, we crossed the gene profiles of the key disease-associated modules from each independent network to identify shared molecular drivers. By intersecting the constituents of the PAD-related Red module with the Sarcopenia-related Pink and Magenta modules, we identified a consensus set of 76 overlapping genes (Figure 4A). KEGG pathway enrichment analysis based on this shared 76-gene set revealed that these genes are mainly enriched in immune and inflammation-related pathways, including classic pathways such as neutrophil extracellular trap formation, chemokine signaling pathways, Fcγ receptor-mediated phagocytosis, and leukocyte transendothelial migration (Figure 4B), suggesting that innate immune responses, represented by neutrophils and macrophages, may play an important role in disease progression. Further GO enrichment analysis revealed that, at the biological process level, these genes are significantly involved in key immune effector processes such as immune response regulation signaling pathways, leukocyte migration, leukocyte adhesion, phagocytosis, and degranulation (Figure 4C). In terms of molecular function, they are mainly involved in phospholipid binding, GTPase regulation activity, and immune receptor activity (Figure 4D), reflecting their important role in cell membrane signal transduction. Regarding cellular components, the proteins encoded by these genes are mainly enriched in structures such as secretory granule membranes, endocytic vesicles, and phagocytic vesicles (Figure 4E), suggesting their close relationship with the secretory and phagocytic functions of immune cells. In summary, these core overlapping genes are mainly involved in immune response processes characterized by innate immune activation, inflammatory cell recruitment, and phagocytosis, and may play a key role in disease-related inflammatory microenvironment remodeling.

### 3.4 Machine learning identifies core genes in PAD and sarcopenia

**FIGURE 5:**
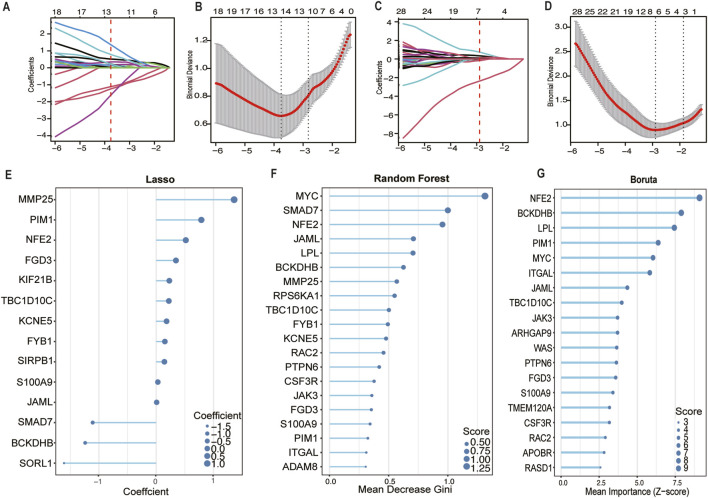
Screening of feature genes using LASSO, random forest, and Boruta algorithms. **(A–D)** LASSO regression feature selection and optimal parameter determination based on ten-fold cross-validation. **(E–G)** Core feature genes identified by LASSO, random forest, and Boruta algorithms, along with assessment of gene importance.

Building upon this foundation, this study further employed three machine learning algorithms—LASSO regression, Random Forest (RF), and Boruta—to perform parallel feature screening of genes from the module intersection, aiming to identify robust core candidate genes. First, LASSO regression was used to reduce the dimensionality of gene features, and the optimal penalty parameter λ was determined through 10-fold cross-validation (Figures 5A–D). Under the optimal λ condition, 13 feature genes were selected (Figure 5E). The Random Forest algorithm identified the top 20 genes with high classification contributions, among which MYC, SMAD7, NFE2, JAML, BCKDHB, and MMP25 showed high importance scores (Figure 5F). The Boruta algorithm confirmed that genes such as NFE2, BCKDHB, LPL, PIM1, MYC, ITGAL, and JAML had stable contributions (Figure 5G). Combining the screening results of the three algorithms, the intersection was used to obtain a set of consensus genes that repeatedly appeared in different models. Finally, five genes—BCKDHB, NFE2, JAML, S100A9, and PIM1—were selected as core candidate biomarkers for subsequent model construction and performance evaluation.

### 3.5 Diagnostic efficacy assessment of key genes

**FIGURE 6:**
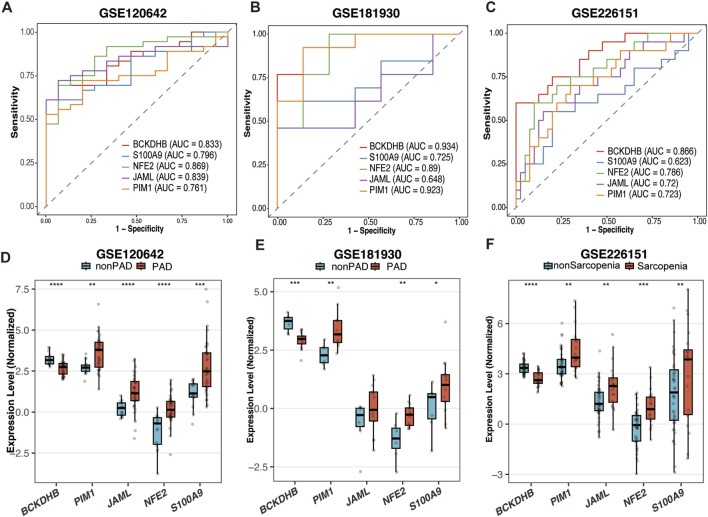
ROC curves of candidate genes in different datasets. **(A–C)** ROC curves for five shared diagnostic markers in GSE120642; GSE181930and GSE226151. **(D–F)** Expression of hub genes in GSE120642; GSE181930and GSE226151.

The discriminative power of key genes selected through machine learning in PAD and its related phenotypes was evaluated. Receiver operating characteristic (ROC) curve analysis was performed on representative genes in the training set GSE120642, the external validation set GSE181930, and the sarcopenia dataset GSE226151. In the GSE120642 training set, BCKDHB, NFE2, JAML, S100A9, and PIM1 all showed good diagnostic efficacy, with NFE2 (AUC = 0.869, 95% CI: 0.767–0.970), JAML (AUC = 0.839, 95% CI: 0.730–0.948), and BCKDHB (AUC = 0.833, 95% CI: 0.723–0.943) showing particularly outstanding discriminative power (Figure 6A). In the independent validation set GSE181930, the above genes generally maintained good discriminative performance. BCKDHB (AUC = 0.934, 95% CI: 0.826–1.000), PIM1 (AUC = 0.923, 95% CI: 0.799–1.000), and NFE2 (AUC = 0.890, 95% CI: 0.723–1.000) showed stable and high diagnostic accuracy, while S100A9 and JAML had relatively lower AUC values, but were still higher than the random classification level (Figure 6B). In the sarcopenia dataset GSE226151, BCKDHB (AUC = 0.866, 95% CI: 0.769–0.963) and NFE2 (AUC = 0.786, 95% CI: 0.663–0.910) maintained good discriminative power, while PIM1, JAML, and S100A9 had moderate AUC values (Figure 6C). Boxplot analysis confirmed significant and consistent baseline expression alterations for these five hub genes across all cohorts (Figures 6D–F). In summary, these key genes demonstrated relatively stable diagnostic efficacy across different datasets and disease contexts, suggesting their potential discriminative value in PAD and its related sarcopenia.

### 3.6 Functional enrichment and interaction network characteristics of key genes

**FIGURE 7:**
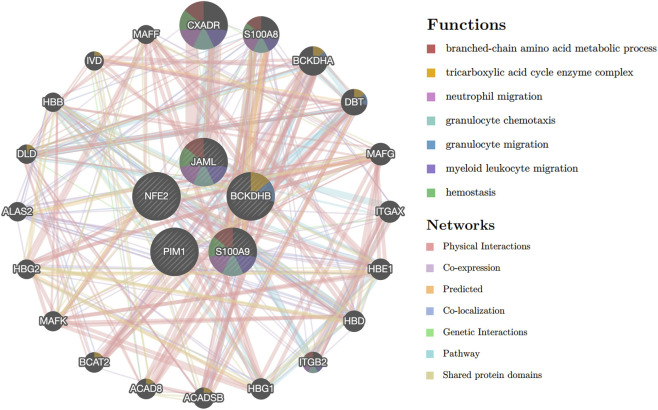
Gene interaction network analysis of candidate genes.

To further explore the functional connections of key genes identified through machine learning screening, a gene-gene interaction network was constructed based on GeneMANIA, and its functional enrichment characteristics were analyzed. As shown in Figure 7, the screened key genes exhibit a highly interconnected structure in the network, with nodes mainly linked through physical interactions, co-expression, predicted interactions, and pathway associations. Functional annotation results indicate that this gene network is significantly enriched in multiple disease-related biological processes, including the tricarboxylic acid cycle enzyme complex, branched-chain amino acid metabolism, reactive oxygen species metabolism, and immune-related pathways such as granulocyte migration, chemotaxis, and myeloid leukocyte migration. Among these, BCKDHB metabolism-related genes mainly participate in the branched-chain amino acid metabolism module, while genes such as S100A8, S100A9, and JAML are concentrated in functions related to granulocyte and myeloid immune cell migration. Notably, some key genes such as BCKDHB, S100A9, and JAML show high connectivity in the network, located at the intersection of metabolic regulation and immune-related functional modules, suggesting that these genes may play a pivotal role between different functional modules. Overall, these key genes form close functional connections in multiple biological processes, including branched-chain amino acid metabolism regulation, oxidative stress, and immune cell migration.

### 3.7 Immune cell infiltration patterns in PAD and sarcopenia

**FIGURE 8:**
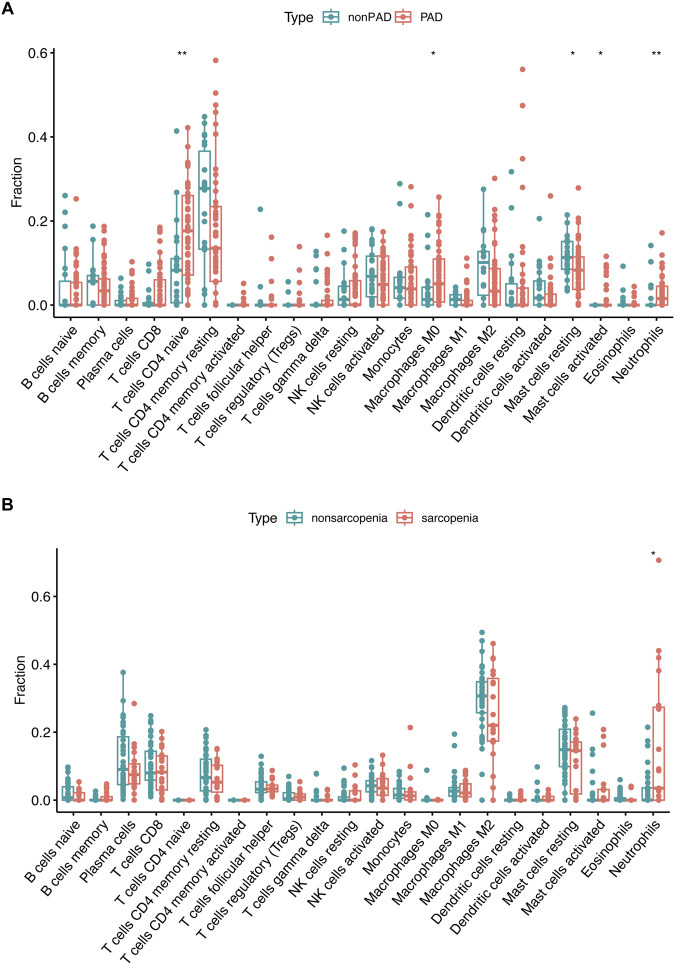
Immune infiltration analysis of PAD and sarcopenia. **(A)** Comparison of immune cell infiltration fractions between the nonPAD and Peripheral Artery Disease (PAD) group. **(B)** Comparison of immune cell infiltration fractions between non-sarcopenia and sarcopenia groups. *P < 0.05, P < 0.01.

To systematically assess changes in the tissue immune microenvironment associated with PAD and sarcopenia, the CIBERSORT algorithm was used to compare the relative proportions of 22 immune cell subsets in the samples. As shown in Figure 8A, compared with the control group, the proportions of multiple immune cell subsets were significantly altered in PAD samples. Specifically, the infiltration proportions of naïve CD4^+^ T cells, Macrophages M0, Macrophages M2, and Neutrophils were significantly increased, while some immune cell subsets showed a decreasing trend in PAD. Overall, PAD tissue exhibited an immune infiltration pattern characterized by an increase in monocytes/macrophages and neutrophils (Figure 8A). In the sarcopenia cohort, the immune cell composition also changed significantly. Compared with non-sarcopenia individuals, the proportion of neutrophils was significantly increased in sarcopenia samples. The consistency between PAD and sarcopenia in neutrophil infiltration characteristics suggests that the two diseases may share similar patterns of change at the immune microenvironment level (Figure 8B).

### 3.8 Correlation analysis of key genes and immune cell infiltration

**FIGURE 9:**
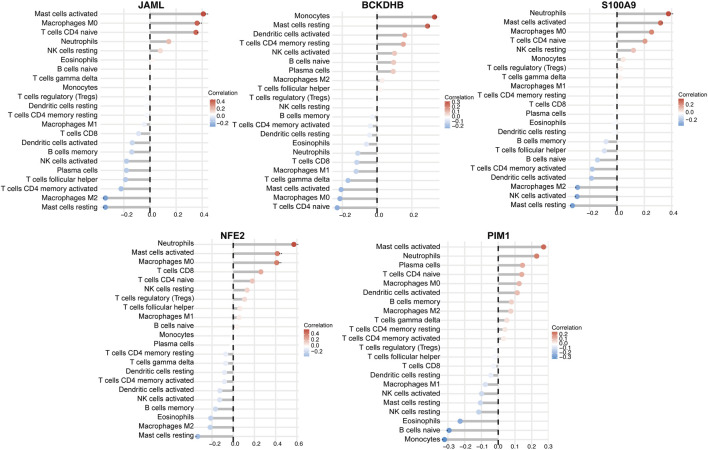
Correlation analysis between feature genes and gene–immune infiltration in PAD.

To further explore the potential association between key genes and the immune microenvironment, Spearman correlation analysis was used to assess the relationship between BCKDHB, NFE2, S100A9, JAML, and PIM1 and the infiltration proportions of 22 immune cell subsets (Figures 9A–E). The results showed that the expression level of S100A9 was significantly positively correlated with various myeloid immune cells, especially Neutrophils, Macrophages M0, and Mast cells activated, while it was negatively correlated with various lymphocyte subsets such as T cells CD4 memory activated, and NK cells activated. The expression of BCKDHB was significantly positively correlated with Monocytes, Mast cells resting, and Dendritic cells activated, while it was negatively correlated with Macrophages M0, T cells CD4 naive, and Mast cells activated. The expression level of NFE2 was significantly positively correlated with Neutrophils, Macrophages M0, and Mast cells activated, while it was negatively correlated with Macrophages M2 and some resting immune cells. Furthermore, JAML expression was positively correlated with Macrophages M0, T cells CD4 naive, and Neutrophils, while PIM1 expression was positively correlated with Mast cells activated, Neutrophils, and Plasma cells, and negatively correlated with Monocytes and B cells naive. Overall, these key genes exhibited consistent correlations with immune cell infiltration, primarily involving myeloid immune cell-related subsets, suggesting their potential involvement in regulating the immune microenvironment characteristics associated with PAD and sarcopenia.

### 3.9 Drug candidates identification based on hub genes

To assess the potential drug targetability of key genes, a systematic drug-gene interaction analysis was performed on the screened core genes using the DGIdb database. The DGIdb interaction score was utilized as a normalized, composite weight derived from the number of peer-reviewed publications supporting the drug-target pair, the target’s specificity, and database-curated validation levels. A higher score reflects enhanced topological closeness and target reliability. The analysis revealed significant heterogeneity in the drug targetability among the core candidate genes (Table 1). S100A9 exhibited the most robust and consistent drug interaction characteristics, with Paquinimod and Tasquinimod showing the highest functional inhibitory interaction scores (Score = 26.102), suggesting high direct translational potential. Additionally, NFE2 was found to interact with the approved drug Paclitaxel (Score = 0.949), suggesting its potential involvement in disease-related transcriptional or oxidative pathways. BCKDHB demonstrated moderate-strength interactions with experimental metabolic compounds. Notably, no drug-gene interaction records were retrieved for JAML within the current DGIdb registry. This indicates that JAML currently lacks established small-molecule modulators, highlighting it as a novel target that warrants future targeted pharmacological investigation.

**TABLE 1:** Core gene-drug interaction score based on DGIdb database.

Gene	Candidate drug(s)	Primary mechanism	Established indication(s)	Interaction score
S100A9	Paquinimod; tasquinimod	S100A8/A9 inhibitor	SLE; mCRPC (Investigated)	26.102
BCKDHB	Seladelpar; lanifibranor	PPAR agonist	PBC (approved); NASH/MASH	1.004
NFE2	Paclitaxel	Microtubule inhibitor	Solid tumors; PAD (device-based)	0.949
PIM1	SEL-24; AZD-1208; PIM447	PIM kinase inhibitor	AML	1.492

Abbreviation: AML, Acute myeloid leukemia; mCRPC, Metastatic prostate cancer; MM, Multiple myeloma; NASH/MASH, Nonalcoholic/metabolic steatohepatitis; PAD, Peripheral artery disease; PBC, Primary biliary cholangitis; PPAR, Peroxisome proliferator-activated receptor; SLE, Systemic lupus erythematosus.

### 3.10 S100A9 causes mitochondrial homeostasis imbalance in skeletal muscle cells

**FIGURE 10:**
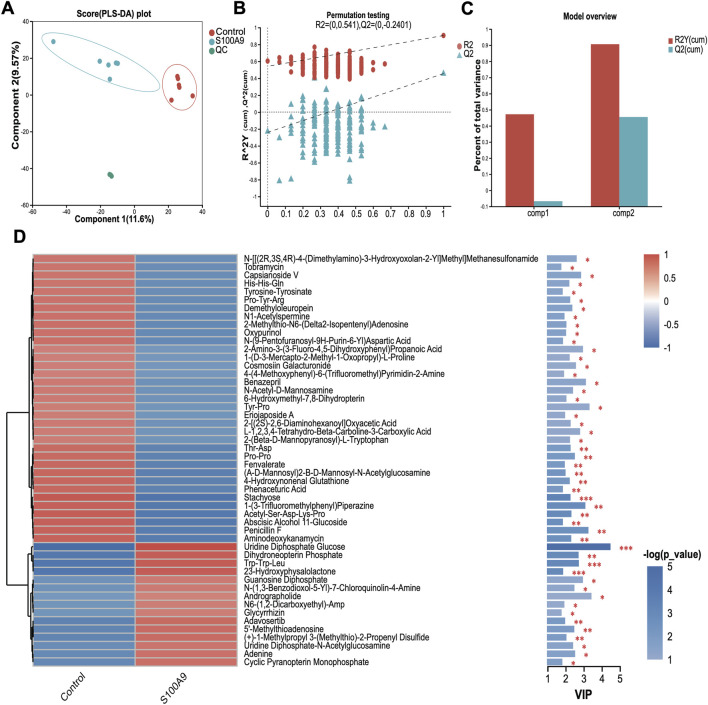
Metabolic alterations in C2C12 cells following rS100A9 treatment. **(A)** PLS-DA score plot showing distinct separation between S100A9 and Control groups. **(B)** Permutation test validating the model robustness (Q2intercept < 0). **(C)** Summary of model fit (R2Y) and predictive ability (Q2). **(D)** Heatmap of top differential metabolites and corresponding VIP scores.

Whether S100A9 has a direct effect on skeletal muscle cells remains unclear. To investigate the direct effect of S100A9 on skeletal muscle cell metabolism, we further examined the changes in the metabolic profile of C2C12 cells after rS100A9 treatment. Figure 10A shows that after dimensionality reduction of the metabolic profiles of the control group and the S100A9-treated group using PLS-DA analysis, the two groups were clearly separated on the principal components, and the QC samples showed good aggregation, indicating that the intervention significantly remodeled the metabolic network, and the data quality was high. The permutation test showed that the Q2 intercept was −0.2401, and the R2 and Q2 of all permutation models were lower than those of the original model, indicating that the model was robust and there was no overfitting (Figures 10B,C). Subsequently, core differentially expressed metabolites were screened based on VIP>1 and p-value. As shown in Figure 10D, rS100A9 treatment significantly downregulated some amino acids and lipid derivatives, while upregulating UDP-Glucose (highest VIP), dihydroxyacetone phosphate, and various purine/pyrimidine nucleotides, suggesting that rS100A9 may intervene in energy metabolism and glyconucleotide synthesis. In the rS100A9 treatment group, several products related to energy sensing and glucose metabolism were significantly upregulated. Among them, UDP-Glucose exhibited an extremely high VIP value (VIP > 4.5) and a highly significant difference (p < 0.001), suggesting that rS100A9 may affect cellular energy reserves by intervening in the glycogen synthesis pathway. Simultaneously, the levels of nucleotide metabolic intermediates such as adenine, 5′-methylthioadenosine, and guanosine diphosphate were significantly increased, reflecting a significant disruption to intracellular nucleotide turnover and energy carrier balance under S100A9-induced stress. 4-Hydroxynonenal glutathione was significantly reduced in the rS100A9 treatment group, which typically indicates the accumulation of lipid peroxidation products and the depletion of endogenous antioxidant chelating capacity. Furthermore, the level of the pro-inflammatory marker dihydroneopterin phosphate was significantly increased, while the level of the anti-inflammatory N1-acetylspermine was significantly downregulated, confirming the potential contribution of rS100A9 to the mitochondrial oxidative stress microenvironment at the metabolic level.

**FIGURE 11:**
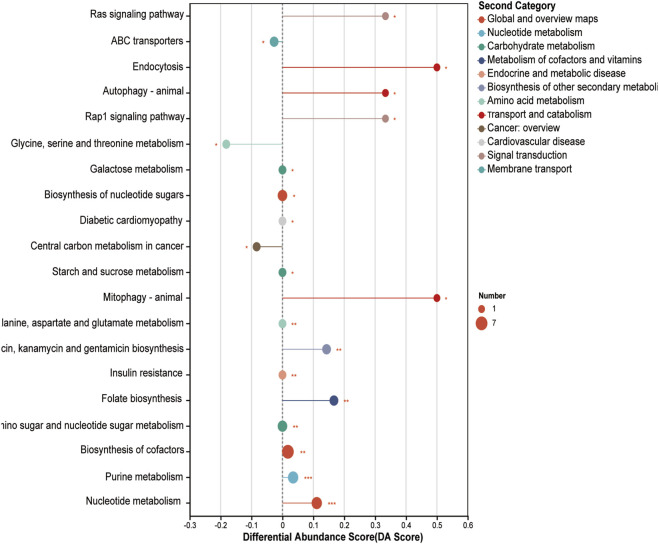
Metabolic pathway enrichment analysis in C2C12 cells after rS100A9 intervention.

To elucidate the macroscopic biological functional changes mediated by these differentially metabolized pathways, we conducted differential abundance score pathway analysis based on the KEGG database (Figure 11). This analysis not only reflects the enrichment level of pathways but also reveals the overall trend of pathway changes. The results showed that nucleotide metabolism and purine metabolism were significantly upregulated after S100A9 intervention, confirming the substantial accumulation of nucleotide derivatives at the microscopic metabolite level. More importantly, mitophagy and its pathway exhibited extremely high positive differential abundance scores. This suggests that S100A9 intervention may mediate cellular oxidative stress response and mitochondrial homeostasis imbalance by triggering mitophagy and endocytosis pathways. Furthermore, amino acid metabolic pathways such as glycine, serine, and threonine were significantly inhibited after S100A9 treatment.

## 4 Discussion

Chronic lower limb ischemia in peripheral artery disease reduces tissue perfusion and drives structural and functional remodeling events in skeletal muscle, including fatty infiltration, fibrosis, muscle fiber atrophy, and fiber-type switching (McDermott, 2015; Hiatt et al., 2015). Clinically, even after technically successful revascularization, some patients show poor recovery of limb function and wound healing, indicating that non-hemodynamic mechanisms play an important role. Sarcopenia, an age-related progressive myopathy defined by loss of skeletal muscle mass, strength, and function, provides a conceptual framework for understanding these non-ischemic contributions. Its core pathologies—chronic low-grade inflammation, mitochondrial dysfunction, and impaired regenerative capacity—are each sufficient to drive muscle degeneration even under non-ischemic conditions. The prevalence of sarcopenia in patients with PAD is approximately 34.6%, substantially higher than that in the general elderly population (Pizzimenti et al., 2020). Moreover, PAD patients with concomitant sarcopenia have higher rates of revascularization failure, amputation, and in-hospital mortality. Sarcopenia is therefore conceptualized not merely as a passive bystander in PAD progression, but as an autonomous pathological determinant shaping the heterogeneity of muscle remodeling and functional recovery.

Our transcriptomic screen identified that BCKDHB, NFE2, and S100A9 are significantly dysregulated in PAD and sarcopenia, suggesting that chronic inflammation, neutrophil dysfunction, and branched-chain amino acid (BCAA) metabolic disorders are core shared pathways. BCKDHB encodes a core catalytic subunit of the rate-limiting branched-chain α-keto acid dehydrogenase (BCKDH) complex (Feige, 2025). Systemically, a landmark community-based study involving over 100,000 UK adults demonstrated that elevated circulating BCAAs are positively correlated with increased muscle mass, with higher circulating valine levels specifically linked to a 47% reduced risk of sarcopenia (Liu et al., 2024). Furthermore, evidence from clinical controlled trials corroborates that targeted nutritional supplementation of BCAAs combined with vitamin D can preserve appendicular lean mass and enhance myocellular mitochondrial bioenergetics and redox activity (Cochet et al., 2023). Paradoxically, however, in clinical cardiology, elevated fasting plasma concentrations of BCAAs are widely recognized as robust, independent biomarkers of insulin resistance, metabolic syndrome, and cardiovascular risk (Ruiz-Canela et al., 2018). Intriguingly, our study reconciles this systemic cardiovascular risk with localized skeletal muscle wasting through the lens of a tissue-specific metabolic mismatch. In PAD, when this elevated systemic amino acid flux encounters an ischemic skeletal muscle bed, the tissue becomes uniquely incapable of processing the metabolic load because the BCKDHB clearance gateway has been systematically downregulated by chronic hypoxia and reactive oxygen species (Neinast M. D. et al., 2019; Ferreira et al., 2023). The resulting local BCKDHB deficiency impairs BCAA breakdown, leading to the pathological intramuscular accumulation of BCAAs and their cytotoxic byproducts, branched-chain α-keto acids (BCKAs) (Holeček, 2018; Neinast M. et al., 2019). This precisely aligns with the recent breakthrough by Zuo et al., which demonstrated that prolonged BCAA/BCKA overaccumulation in sarcopenic muscle overactivated local mTORC1 signaling, which paradoxically arrests autophagic flux, disrupts mitochondrial oxidative phosphorylation, and induces myofiber atrophy (Zuo et al., 2025). This convergence positions defective BCAA catabolism as a shared metabolic crossover point between PAD and sarcopenia and suggests that the therapeutic strategy in this population should target restoration of muscle-autonomous BCAA catabolic capacity rather than systemic BCAA supplementation alone.

Our study revealed that S100A9 is significantly overexpressed in the skeletal muscle of PAD and sarcopenia patients. Although contracting skeletal muscle fibers themselves have been reported to express and release S100A8/A9 under physiological conditions (Mortensen et al., 2008), S100A9 primarily functions as a myeloid-derived damage-associated molecular pattern (DAMP), which is secreted into the muscle interstitium by infiltrating neutrophils and monocytes and exerts its effects through paracrine signaling (Zhang et al., 2026). Mechanistically, recent work by Huang et al. demonstrated that in a murine sepsis model, S100A9 binds to the RAGE receptor in skeletal muscle, induces Drp1 phosphorylation, and drives excessive mitochondrial fission and fragmentation (Wang et al., 2026; Huang et al., 2025).Notably, recombinant S100A8/A9 directly impairs mitochondrial oxidative phosphorylation and induces myotube atrophy even under normoxic culture conditions, excluding the confounding influence of hypoxia and establishing a direct, cell-autonomous effect on muscle cells (Salyers et al., 2022). These observations, established in sepsis and in normoxic culture, provide a mechanistic framework for interpreting the role of S100A9 in the distinct but overlapping context of chronic ischemic muscle disease (Wang et al., 2024). In this study, treatment of C2C12 myoblasts with rS100A9 mimicked the sensitivity of muscle progenitor cells to stress signals from infiltrating mesenchymal myeloid cells. Metabolomics analysis confirmed that rS100A9 disrupted nucleotide turnover and energy carrier balance, manifested as elevated levels of adenine and guanosine diphosphate. More importantly, we observed significant upregulation of mitophagy and endocytosis pathways, indicating an imbalance between cellular oxidative stress response and mitochondrial homeostasis. While mitophagy initially begins as an adaptive attempt to clear damaged organelles, our data suggest that under sustained S100A9 stress, nucleotide metabolic disturbances and depletion of endogenous antioxidants, such as 4-hydroxynonenal glutathione, imply that S100A9 leads to a severe intracellular energy crisis. This suggests that dysregulated mitophagy, instead of restoring homeostasis, actively contributes to myocyte bioenergy depletion. Overall, these findings are consistent with the research of Huang et al. and Salyers et al. (Salyers et al., 2022; Wang et al., 2024). Beyond its direct mitochondrial effects, S100A9 serves as a key regulator of macrophage polarization and inflammatory signaling within ischemic microenvironments. Ganta et al. demonstrated that S100A8/A9 acts as a crucial downstream mediator of the anti-angiogenic VEGF165b–VEGFR1 signaling pathway in macrophages (Ganta et al., 2019). Specifically, ischemia-induced VEGF165b blocks VEGFR1, triggering an S100A8/A9-dependent calcium influx that drives pro-inflammatory M1 polarization, thereby impairing neovascularization and perfusion recovery (Ganta et al., 2019). Extracellularly, S100A9 binds macrophage TLR4 or RAGE to activate NF-κB signaling, establishing a feed-forward inflammatory loop that drives the hypersecretion of TNF-α, IL-1β, and IL-6, which inhibit satellite cell myogenic differentiation and impair angiogenesis (Yuan et al., 2026).

In the present study, neutrophil infiltration was identified as a shared immune feature of both PAD and sarcopenia. Persistent neutrophil accumulation may contribute to chronic sterile inflammation through excessive release of reactive oxygen species, proteases, and NET-associated inflammatory mediators, thereby impairing muscle fibers and disrupting the satellite cell microenvironment (Papayannopoulos, 2018). Notably, we observed increased expression of NFE2 in ischemic skeletal muscle, suggesting the presence of altered myeloid-associated transcriptional programs under chronic ischemic stress (Ma et al., 2025). Although our immune infiltration deconvolution utilized the peripheral blood-derived CIBERSORT LM22 matrix (Newman et al., 2015), which may carry inherent biases due to the low baseline abundance of immune cells and complex stroma in skeletal muscle tissue, the identified myeloid-driven features are strongly backed by recent high-resolution tissue-specific data. High-throughput single-cell transcriptomic atlases of chronic limb-threatening ischemia and severe muscle wasting models have unequivocally confirmed massive local myeloid lineage shifts and persistent neutrophil and macrophage accumulation in diseased or ischemic skeletal muscle microenvironments (Cho et al., 2020; Guan et al., 2022; Liu et al., 2025). These single-cell observations strongly cross-validate our computational findings, confirming that neutrophil accumulation is a reliable pathophysiological hallmark of ischemic muscle degeneration.

DGIdb screening identified Paquinimod and Tasquinimod as candidate modulators of the S100A9 axis, though their clinical applicability varies substantially based on their off-target profiles. Paquinimod, a highly selective oral S100A9 inhibitor with an established Phase II clinical safety record in inflammatory diseases (Hesselstrand et al., 2021), represents a viable candidate for therapeutic repositioning to attenuate myeloid-driven inflammation in the PAD. In contrast, Tasquinimod exhibits off-target binding to HDAC4 and upregulates thrombospondin-1 (Olsson et al., 2010; Isaacs et al., 2013). Although its anti-angiogenic activity is therapeutically advantageous in oncology (Sternberg et al., 2016), suppressing angiogenesis in PAD patients could hinder collateral vessel sprouting and capillary growth in ischemic limbs, potentially exacerbating peripheral perfusion deficits. Consequently, highly selective S100A9 inhibition, rather than broad-spectrum targeting, represents a more clinically viable strategy for this comorbidity.

This finding acquires relevance, however, when contextualized within current endovascular practice: Paclitaxel is delivered locally via drug-coated balloons or drug-eluting stents to prevent post-angioplasty restenosis. Beyond its established anti-restenotic role, this drug-gene pair offers a molecular perspective on the clinical benefit of Paclitaxel-coated devices (Tepe et al., 2015). NFE2 regulates megakaryocyte maturation and myeloid-associated transcriptional programs under chronic ischemic stress (Gasiorek and Blank, 2015; Shivdasani et al., 1995). The Paclitaxel-NFE2 axis therefore suggests that localized, device-delivered Paclitaxel within the arterial wall may extend its therapeutic reach downstream—potentially modulating microvascular thrombosis and endothelial-driven remodeling in the adjacent skeletal muscle parenchyma without eliciting systemic cytotoxicity.

## 5 Limitations

Several limitations of the present study should be acknowledged. First, our transcriptomic findings were derived from publicly available retrospective cohorts with relatively modest sample sizes; prospective validation in larger, multi-center clinical cohorts is therefore needed. Second, the immune infiltration analysis was executed using the CIBERSORT LM22 signature matrix. Because the LM22 reference panel is predominantly designed for peripheral blood or immune-cell-enriched tissues, applying it to skeletal muscle may introduce algorithmic bias and limit the resolution of tissue-resident immune phenotypes. Finally, our metabolomic validation used undifferentiated C2C12 myoblasts rather than differentiated myotubes or *in vivo* models. Although lacking the contractile and metabolic microenvironment of mature muscle, this progenitor model uniquely captured early-stage metabolic stress and S100A9-induced mitochondrial dysfunction, which are directly relevant to the regeneration failure in PAD and sarcopenia. Future validation in differentiated myotubes and *in vivo* systems is warranted to substantiate these findings.

## 6 Conclusion

This study explored the common molecular mechanisms of PAD and sarcopenia based on comorbidity analysis. The results indicate that upregulation of skeletal muscle inflammation and abnormal branched-chain amino acid metabolism may be common diagnostic pathways for both diseases. We identified BCKDHB, PIM1, JAML, NFE2, and S100A9 as common diagnostic biomarkers for both diseases. Increased granulocyte infiltration is a shared immune cell pattern in both diseases. Furthermore, metabolomics analysis further confirms that S100A9 may be a potential intervention target.

## Data Availability

The original contributions presented in the study are publicly available. This data can be found in the Gene Expression Omnibus repository with the accession numbers GSE120642, GSE181930, and GSE226151.
